# KLF13 promotes esophageal cancer progression and regulates triacylglyceride and free fatty acid metabolism through GPIHBP1

**DOI:** 10.1038/s41419-025-07709-7

**Published:** 2025-05-31

**Authors:** Pengjie Yang, Benben Zhu, Hongwei Cui, Yongjun Yu, Qin Yu, Linghui Kong, Mengfei Sun, Yuan Liu, Bateer Han, Shuchen Chen

**Affiliations:** 1 Department of Thoracic Surgery, Fujian Medical University Union Hospital, Fuzhou, Fujian Province P.R. China; 2 Department of Thoracic Surgery, Peking University Cancer Hospital (Inner Mongolia Campus)/Affiliated Cancer Hospital of Inner Mongolia Medical University, Hohhot, Inner Mongolia Autonomous Region P.R. China; 3 Clinical Research Center for Thoracic Tumors of Fujian Province, Fuzhou, Fujian Province P.R. China; 4 Department of Pharmacy, Peking University Cancer Hospital (Inner Mongolia Campus)/Affiliated Cancer Hospital of Inner Mongolia Medical University, Hohhot, Inner Mongolia Autonomous Region P.R. China; 5 Scientific Research Department, Peking University Cancer Hospital (Inner Mongolia Campus)/Affiliated Cancer Hospital of Inner Mongolia Medical University, Hohhot, Inner Mongolia Autonomous Region P.R. China; 6 Department of Thoracic Surgery, Inner Mongolia Medical University, Hohhot, Inner Mongolia Autonomous Region P.R. China; 7 Department of Cardio-Thoracic Surgery, The Second Hospital of Chifeng, Chifeng, Inner Mongolia Autonomous Region P.R. China; 8 Department of Radiotherapy, Peking University Cancer Hospital (Inner Mongolia Campus)/Affiliated Cancer Hospital of Inner Mongolia Medical University, Hohhot, Inner Mongolia Autonomous Region P.R. China; 9 Department of Pathology, Peking University Cancer Hospital (Inner Mongolia Campus)/Affiliated Cancer Hospital of Inner Mongolia Medical University, Hohhot, Inner Mongolia Autonomous Region P.R. China; 10 College of Pharmacy, Inner Mongolia Medical University, Hohhot, Inner Mongolia Autonomous Region P.R. China; 11 Department of integrated Chinese and Western medicine, Inner Mongolia Mental Health Center, Third Hospital of Inner Mongolia Autonomous Region, Hohhot, Inner Mongolia Autonomous Region P.R. China; 12 Key Laboratory of Ministry of Education for Gastrointestinal Cancer, Fujian Medical University, Fuzhou, Fujian Province P.R. China; 13 Fujian Provincial Key Laboratory of Cardiothoracic Surgery, Fujian Medical University Union Hospital, Fuzhou, Fujian Province P.R. China; 14 Department of Operation, Fujian Medical University Union Hospital, Fuzhou, Fujian Province P.R. China

**Keywords:** Cancer metabolism, Cell growth

## Abstract

Kruppel-Like Factor 13 (KLF13) has strong effects on cancer occurrence and progression. Nevertheless, the role of KLF13 in oesophagal cancer (EC) remain elusive. In this study, we detected the expression of KLF13 in EC tissues and cells using immunohistochemistry, western blot, and real-time PCR, and found that KLF13 was upregulated in EC tissues and cells compared to normal controls. High expression of KLF13 indicated a poor prognosis for EC patients. Further, function studies in vitro and in vivo were performed to explore the role of KLF13 in EC cell progression. The results revealed that KLF13 knockdown suppressed EC cell proliferation, migration, epithelial-mesenchymal transition, increased cell apoptosis and cell cycle arrest in vivo and inhibited tumour growth in vitro. Conversely, KLF13 overexpression in EC cells had the opposite consequences. Mechanically, differentially expressed genes downstream of KLF13 were identified by RNA-seq and ChIP-seq. We found that there is a positive correlation between triacylglyceride and free fatty acid levels and KLF13 expression levels. A lipid-related gene, Glycosylphosphatidylinositol anchored high density lipoprotein binding protein 1 (GPIHBP1), was identified as a downstream gene of KLF13 using luciferase and chromatin immunoprecipitation assays, whose expression was positively regulated by KLF13. Finally, in vitro and in vivo recovery assays using shRNAs and overexpression plasmids confirmed that KLF13 has an oncogenic role in EC progression through GPIHBP1. Collectively, KLF13 can promote EC progression, triacylglyceride and free fatty acid metabolism through GPIHBP1. Therefore, molecular therapies targeting KLF13 and GPIHBP1 may be effective treatments against EC.

## Introduction

Esophageal cancer (EC), the sixth major cause of cancer-associated mortality across the world [1], can be classified into adenocarcinoma (EAC) and squamous cell carcinoma (ESCC). Although there is considerable overlap between treatments for EAC and ESCC, differences in their molecular characteristics suggest that therapies should differ [2]. Despite recent advances in therapeutics against ESCC, the 5-year survival rate varies from 9% to 27.1% [3–5]. Therefore, there is a high demand for the identification of novel therapeutic targets.

The Kruppel-Like Factor (KLF) family of transcription regulatory proteins has 18 members; these zinc finger proteins control the activation and suppression of transcription through binding to DNA, RNA, and proteins [6]. KLFs can be divided into three subgroups due to molecular phylogeny [7], and have key roles in development, differentiation, and other physiological and cellular processes [8].

KLFs are also established as playing important roles in human cancer. In EC, KLF2 [9] and KLF6 [10] mediate oncogenic function, whereas KLF3 [11], KLF4, KLF5 [12], KLF9 [13], and KLF17 [14] have tumor suppressor roles. KLF10 and KLF15 can regulate LCN2 transcription to control innate immunity in ESCC [15]. Genome-wide analysis detected KLF12 amplification in ~40% of EAC [16]. KLF13 has significant effects on growth and other physiological processes in human cancers [17–19]; however, there are no reports to date of functional effects of KLF13 in EC.

In this study, we identified a novel and highly expressed KLF13 in EC patient samples. We further found that KLF13 could promote EC cell proliferation and migration. Furthermore, KLF13 directly bound to GPIHBP1 and regulated its transcription in EC cells. All these findings suggest that KLF13/GPIHBP1 signalling is crucial for the progression of EC and may be a promising therapeutic target for EC.

## Materials and methods

### Samples from The Cancer Genome Atlas (TCGA) and clinical patients

KLF13 expression in tumour and normal tissue samples was analysed using the ESCA and GSE45670 datasets from TCGA and clinical patients (100 EC and 80 adjacent normal tissue samples from patients undergoing esophagostomy, without chemotherapy or radiotherapy before operation). This study was in accordance with the Declaration of Helsinki and approved by the ethics committee of Peking University Cancer Hospital/Affiliated Cancer Hospital of Inner Mongolia Medical University (No. KY202404).

### Cell culture and infection

The human EC cell line, TE-1, was obtained from the cell bank of the Shanghai Institute for Biological Sciences (1101HUM-PUMC000986). KYSE30 (CL-0577), KYSE150 (CL-0638) and KYSE180 (CL-0760) cells were obtained from Procell Life Science & Technology. KYSE450 was obtained from the iCell Bioscience Inc. (iCell-h494). KYSE70 was obtained from the Shanghai Enzyme-linked Biotechnology Co., Ltd (ml056342). Cells were cultured in RPMI 1640 medium (Hyclone), which contains 10% fetal bovine serum (Gibco) and 1% penicillin/streptomycin (Hyclone), and kept in a 37 °C incubator with 5% CO_2_. KLF13 shRNA lentiviral particles (shKLF13#1 and shKLF13#2), shGPIHBP1, shCtrl and KLF13 overexpression lentiviral particles were purchased from Genechem (Shanghai, China). Briefly, after cloning the shRNA into the pGCSIL-GFP vectors, 15 μg of pGCSIL-GFP-KLF13 or shGPIHBP1 -1/2 vectors were co-transfected with 5 μg of pHelper1.0 and 5 μg of Helper2.0 into 293FT cells using Invitrogen™ Lipofectamine™ 2000 (Thermo Fisher Scientific, Inc.). The whole open reading frame of porcine *KLF13* was inserted into pcDNA3.1 (Invitrogen) to generate a KLF13 overexpression vector (GV341-KLF13), and transfected into 293FT cells using Lipofectamine 2000. Then, the Viral supernatants were harvested for subsequent infection according to the manufacturer.

### Immunohistochemistry (IHC)

The paraffin-embedded sections were deparaffinized, rehydrated, and incubated with 3% H_2_O_2_ for 3 mins. Next, phosphate-buffered saline containing 0.3% Triton was used to permeabilize sections for 15 min, followed by blocking in 3% bovine serum albumin solution for 1 h. Then, sections were incubated with primary antibody against KLF13 (PLLABS, PL0303771, 1:200) at 4 °C overnight and secondary antibody at 25 °C for 1 h. The sections were visualized using a DAB chromogenic kit (Servicebio), and counterstaining with hematoxylin.

### Quantitative real-time PCR assays

An RNAqueous®-4PCR kit was adopted for total RNA extraction from EC samples and cells. For quantitative PCR, 2*SYBR Green qPCR Master Mix (Low ROX) (Servicebio) were conducted using the Applied Biosystems 7500 Real-Time PCR System with the following conditions: 95 °C for 5 s, 55 °C for 30 s, 72 °C for 30s for 40 cycles. The following primers were used: *KLF13*, forward, 5’-CGGCCTCAGACAAAGGGTC-3’, reverse, 5’-TTCCCGTAAACTTTCTCGCAG-3’; *GPIHBP1*, forward, 5’-GCAACCTGACGCAGAACTG-3’, reverse, 5’-CCAGGGTGGGACATTGCAC-3’; β-actin, forward, 5’-CATGTACGTTGCTATCCAGGC-3’, reverse, 5’-CTCCTTAATGTCACGCACGAT-3’. All qPCR assays were conducted in triplicate. Data were analysed using the comparative CT method.

### Western blot

EC cells were lysed (on ice) in RIPA lysis buffer (Beyotime). The concentration of the protein was determined by BCA kit (Beyotime). A total of 30 μg protein lysates were subjected to sodium dodecyl sulfate-polyacrylamide gel electrophoresis and transferred to polyvinylidene difluoride membranes (Millipore, Darmstadt, Germany). After blocking in 5% nonfat milk at 25 °C for 1 h, the membranes were incubated with primary antibodies against: KLF13 (Invitrogen, PA1-4117), E-cadherin (Invitrogen, 14-3249-82), N-cadherin (Invitrogen, MA5-32088), caspase 3 (CST, #9665), cleaved caspase 3 (cle-caspase 3) (CST, #9661), or GPIHBP1 (Invitrogen, PA5-98598), at 4 °C with a 1:1000 dilution. HRP-conjugated secondary antibodies (1:5000) were added for 1 h of incubation at 25 °C. A Western Lightning™ Chemiluminescence Reagent Plus kit was used to visualise proteins, and analysed using ImageJ software (National Institutes of Health). GAPDH (Invitrogen, MA5-15738) served as the internal control.

### Cell proliferation assay

A cell Counting Kit-8 (Beyontin, Shanghai, China) was used for EC cell viability. For cell colony formation assays, a total of 500 transfected EC cells were plated into 6-well plates per well. 4% paraformaldehyde was used to fix the colonies for 30 min after 14-day incubation, followed by staining 15 min with 0.1% crystal violet solution (Sigma, USA). Colonies above 50 cells were counted under microscopy.

### Cell migration assay

Migration assays were conducted using 8 µm transwell filters (Corning). EC cells (5 × 10^4^) in 100 µl serum-free medium were added to the upper chambers. Medium (600 µl) containing 20% FBS (as a chemoattractant) was injected into the lower chambers. After 24 h incubation at 37 °C with 5% CO_2_, cells were fixed in 4% paraformaldehyde, followed by staining with 0.1% crystal violet solution, and image capture by photography.

For wound-healing assays, EC cells were cultured in six-well plates, and a linear wound was made in the cell monolayers by scraping with a sterile pipette tip. Cell Images were captured 24 and 48 h after scratching. ImageJ software was used to measure the distance between the two edges of the scratched wound.

### Flow cytometry analysis

An Annexin V-FITC/PE apoptosis detection kit was adopted to analyze apoptosis according to the manufacturer’s instructions. For cell cycle analysis, transfected EC cells were fixed in 70% ethanol at −20 °C overnight, followed by staining with DNA staining solution containing PI and RNaseA (Sigma-Aldrich). LSRII cytometry (BD Biosciences) was used to obtain data, followed by analysis using FlowJo software (FlowJo, Ashland, OR).

### Animal experiments

Twenty-five male BALB/c-nude mice (6-week-old), provided by Vital River (Beijing), were randomly divided into five groups (shCtrl, shKLF13; Ctrl, KLF13, KLF13+shGPIHBP1, *n* = 5), followed by subcutaneous injection with KYSE450 cells or KYSE30 cells (6 × 10^5^ cells per mouse) into the right flank area. After visible tumors developed, tumor volume was calculated as length × width^2^/2 every 3 days. After 35 days, tumors were dissected, weighed, and stained for IHC analysis. This work was carried out according to the *Basel Declaration* and approved by the ethics committee of Peking University Cancer Hospital/Affiliated Cancer Hospital of Inner Mongolia Medical University (No. KY202404).

### RNA sequencing and gene set enrichment analysis (GSEA)

Trizol reagent was used to extract RNA from control (Ctrl) and shKLF13-transfected ESSC cells. A Qubit RNA fluorometer (Invitrogen) was used to quantify RNA, and an Agilent Bioanalyzer 2100 was employed to evaluate RNA integrity. Libraries were generated using Illumina TruSeq stranded mRNA chemistry, applying Micromon (Monash University, Melbourne, Australia). An Illumina NextSeq500 in High-Output mode was used to sequence the libraries. In addition, GSEA [20] was applied to explore the pathways and functional mechanisms underlying differential gene enrichment in the group with low KLF13 expression.

### Metabolomics analysis

Infected EC cells were harvested, washed, and cell pellets quenched in 60% methanol containing 0.85% (wt/vol) ammonium bicarbonate at −40 °C. Then, two 100% methanol extractions were used to extract the metabolites from quenched cells, followed by a single water extraction [21]. Metabolite samples were then transferred to Metabo-Profile Biotechnology (Shanghai) Co., Ltd for metabolomics analysis. The project was performed under the guidance of Quality Management System ISO 9001:2015 (QAIC/CN/170149).

### RNA-seq analysis

Differential expression analysis of metabolic genes was conducted using linear model RNA-seq analysis software [22]. Genes were regarded as differentially expressed when *p* < 0.05 and log_2_ variation was >2-fold. A heatmap was produced using R software [23].

### ChIP-seq promoter analysis

ChIP-seq promoter analyses were performed using a Novo NGS® CUT & Tag High-Sensitivity Kit (Novo protein, N259-YH01) according to the manufacturer’s instructions. Sequencing was performed using the Nova Seq platform, and promoters bound by KLF13 were screened using IGV software [24].

### Chromatin immunoprecipitation (ChIP)-PCR

ChIP assay kits (Millipore-Sigma, EZ-ChIP, Cat: 17-371) were used in this study according to the manufacturer’s instructions. ChIP signals were calculated as % input = 1% × 2^(CT input – CT sample)^.

### Luciferase assay

Dual luciferase assay kits (Promega) were used to conduct the luciferase assay. The recombinant plasmids containing the *GPIHBP1* promote-Luc/Rluc were constructed and transfected into pretreated KYSE450 cells with ectopic Flag-KLF13 expression and their control cells. A microplate reader (Flexstation® 3, Molecular Devices (MD), USA) was used to detect fluorescence intensity. Transfection efficiency was normalized using an internal control of pRL-null vector expressing Renilla luciferase. Kruskal–Walli’s test was adopted to analyze the differences.

### Triacylglyceride (TAG) and free fatty-acid (FFA) measurement

Triacylglyceride levels were quantified using the Triglyceride Assay Kit (Abcam, #ab65336) according to the manufacturer’s instructions [25]. Briefly, 1 × 10^7^ cells were harvested and washed with cold PBS, and subsequently resuspended in 500 μl of 5% NP-40 solution. After two cycles of heating at 95 °C for 5 min followed by cooling to room temperature to solubilise all triglycerides, the samples were diluted in water and mixed with cholesterol esterase/lipase for 20 min. Then, the triglyceride reaction mix was added and incubated for 60 min at room temperature. The intensity of OD570 nm was measured using a Synergy 2 microplate reader (BioTek). A free fatty-acid fluorometric assay kit (ab65341, Abcam) was used to measure FFA levels according to the former report [26]. Experiments were conducted using triplicate biological samples to ensure reproducibility.

### Statistical analysis

Quantitative data are presented as mean ± SD values, and GraphPad Prism Version 9.0 was used for statistical analyses. Differences between the two groups were assessed by Student’s t test. Correlations were conducted by calculating Pearson’s *r* values. Results from in vitro assays are presented for three independent experiments. *P* < 0.05 was considered significant.

## Results

### KLF13 is highly expressed in EC

**Fig. 1:**
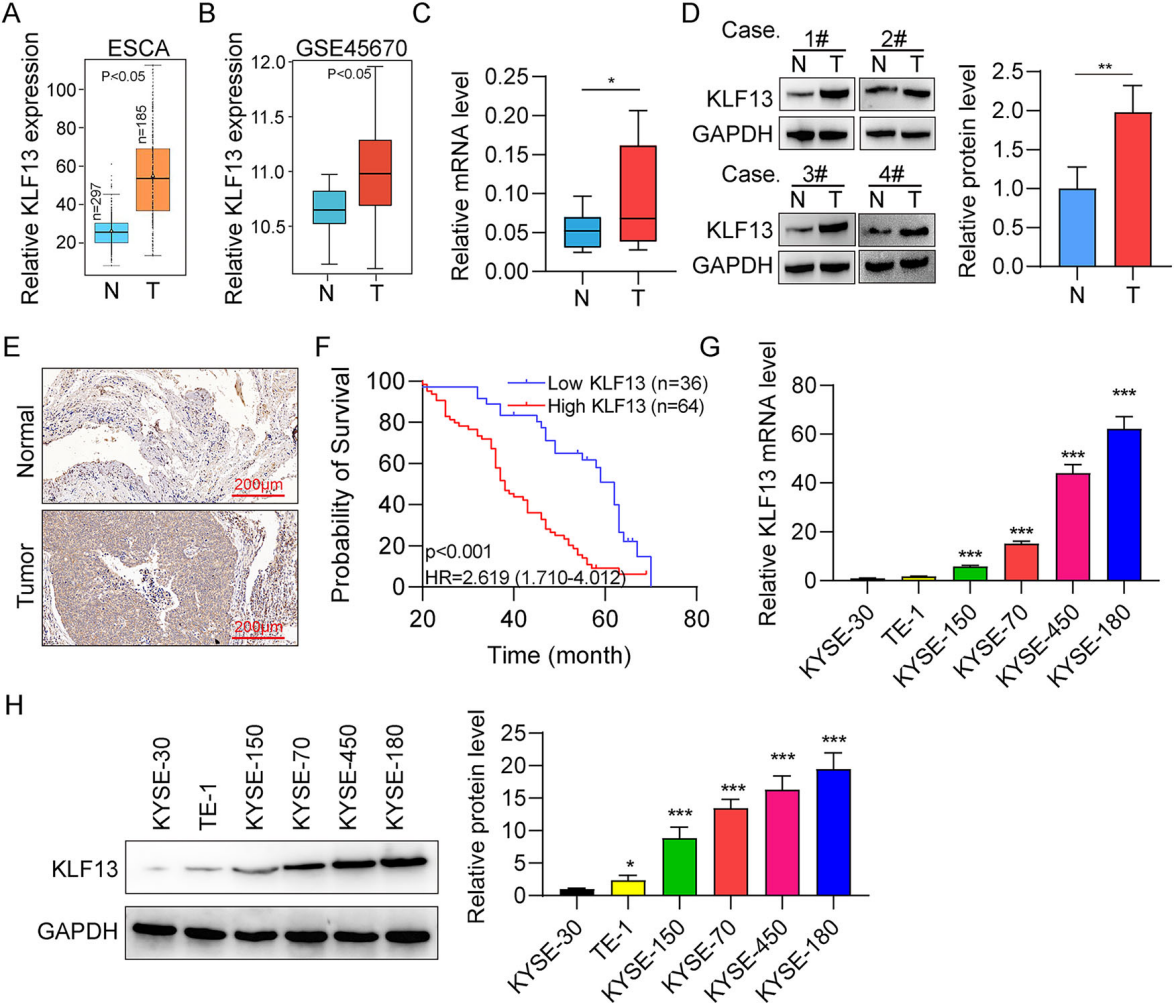
KLF13 is upregulated in EC. **A**, **B** *KLF13* mRNA levels in EC samples and normal controls from the TCGA database ESCA and GSE45670 datasets. **C**, **D** Comparison of KLF13 transcript and protein levels in EC tumors and normal control samples. **E** Representative KLF13 IHC staining in EC tissues and normal ones. Scale bar, 200 µm. **F** Survival analysis according to KLF13 expression level in EC using Kaplan-Meier Plotter. **G**, **H** KLF13 transcript and protein levels in EC cell lines.

First, *KLF13* expression was analyzed using the ESCA and GSE45670 datasets by Gene Expression Profiling Interactive Analysis [27]. *KLF13* was upregulated in EC tissue than in normal tissue samples (Fig. 1A, B). Next, KLF13 expression in EC and adjacent non-cancerous tissues was evaluated by RT-qPCR and western blot. Upregulated KLF13 levels were found in tumor tissues relative to those in adjacent non-cancerous specimens (Fig. 1C, D). Further, IHC staining also demonstrated higher KLF13 expression in EC tumors than in adjacent non-cancerous tissue (Fig. 1E, Table 1). Analysis using Kaplan–Meier plotter demonstrated that high *KLF13* mRNA expression showed shorter patient survival (Fig. 1F) and tumor size (Table S1). Moreover, KLF13 expression in four EC cells (KYSE-70, KYSE-150, KYSE-180, KYSE-450) were markedly greater than KYSE-30 and TE-1 cells (Fig. 1G, H). These results show that KLF13 expression levels are upregulated in EC.

**Table 1:** The protein abundance of KLF13 in ESCC by IHC.

	Normal	Cancer	*χ*^2^	*p* value
High expression	27	64	16.27	<0.001
Low expression	53	36		
Total	80	100		

### Silencing KLF13 suppressed EC cell proliferation and migration

**Fig. 2:**
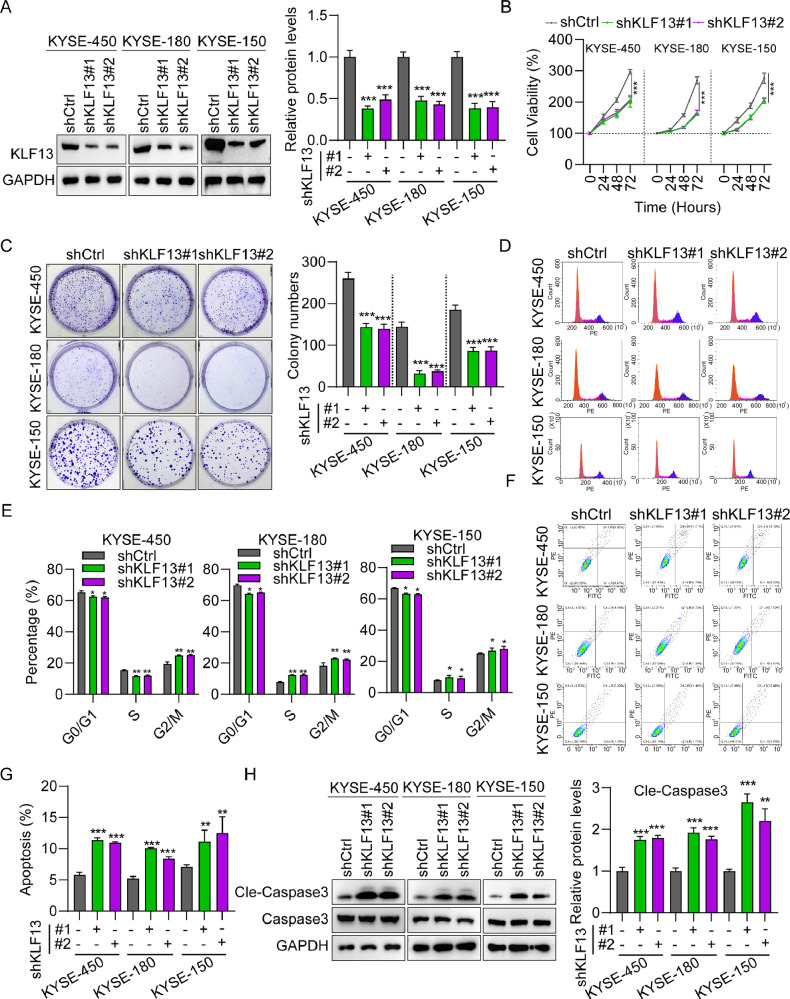
KLF13 silencing suppressed EC cell proliferation. **A** KLF13 expression in KYSE-450, KYSE-180 and KYSE-150 cells infected with shKLF13#1, shKLF13#2, and shCtrl. **B** Cell viability of EC cells after KLF13 knockdown. **C** Cell colony formation assay to determine the effect of KLF13 knockdown on EC cell proliferation. **D**, **E** Cell cycle distribution of two EC cells infected with shCtrl, shKLF13#1 and shKLF13#2, assessed by flow cytometry. **F**, **G** Apoptosis of EC cells transfected with shKLF13#1, shKLF13#2, and shCtrl, assessed by flow cytometry. **H** Caspase 3 and cle-caspase 3 protein levels in EC cells infected with shKLF13#1, shKLF13#2, and shCtrl.

Next, KLF13 stably knocked down were established in KYSE-450, KYSE-180 and KYSE-150 cells using KLF13-specific shRNA; and the knockdown efficiency was confirmed using western blot (Fig. 2A). KLF13 knockdown resulted in an approximately 30% decline in cell viability (Fig. 2B), and a decreased in cell colony formation of around 50% (Fig. 2C). Flow cytometry analysis demonstrated that KLF13 knockdown decreased the proportions of KYSE-450, KYSE-180 and KYSE-150 cells in G0/G1 (Fig. 2D, E) and greatly increased apoptosis ratio of KYSE-450, KYSE-180 and KYSE-150 cells (Fig. 2F, G). KLF13 knockdown also increased the cle-caspase 3 protein levels in KYSE-450, KYSE-180 and KYSE-150 cells (Fig. 2H). Furthermore, KLF13 knockdown greatly suppressed EC cell migration (Fig. S1A, B) and promoted E-cadherin protein expression and suppressed that of N-cadherin, while KLF13 overexpression had the opposite outcomes (Fig. S1C). These results indicated that KLF13 knockdown can suppress EC cell proliferation and migration.

### Overexpression of KLF13 promoted EC cell proliferation and migration

**Fig. 3:**
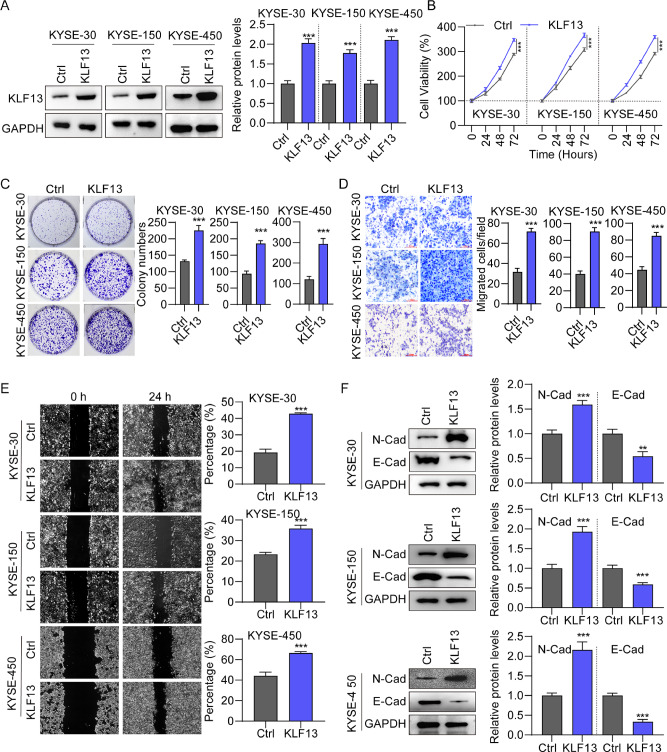
Overexpression of KLF13 promotes esophageal cancer proliferation and metastasis. **A** KLF13 expression in EC cells infected with KLF13 and Ctrl goups. **B** Cell viability of EC cells following KLF13 overexpression. **C** Cell colony formation assay to determine the effect of KLF13 overexpression on EC cell proliferation. **D**, **E** Transwell and wound-healing assays to examine migration of EC cells transfected with KLF13 and Ctrl. **F** The protein levels of E-cadherin and N-cadherin in EC cells following KLF13 overexpression.

Next, KLF13 overexpression lentiviurs were used to evaluate the effect of endogenous KLF13 on EC progression. We found that KLF13 expression was upregulated in KYSE-30, KYSE-150 and KYSE-450 cells after KLF13 overexpression (Fig. 3A). CCK-8 (Fig. 3B) and cell colony formation (Fig. 3C) assays results revealed that ectopic KLF13 expression greatly increased the ability of cell proliferation compared to the control group. We also found that KLF13 overexpression resulted in markedly increased cell migration in transwell (Fig. 3D) and wound-healing (Fig. 3E) assays, relative to controls. Examination of EMT biomarker expression demonstrated that N-cadherin levels were elevated, while those of E-cadherin were reduced in cells overexpressing KLF13 (Fig. 3F). These outcomes further indicate the oncogenic role of KLF13 on EC cell progression.

### Silencing KLF13 suppressed EC tumor growth in vivo

**Fig. 4:**
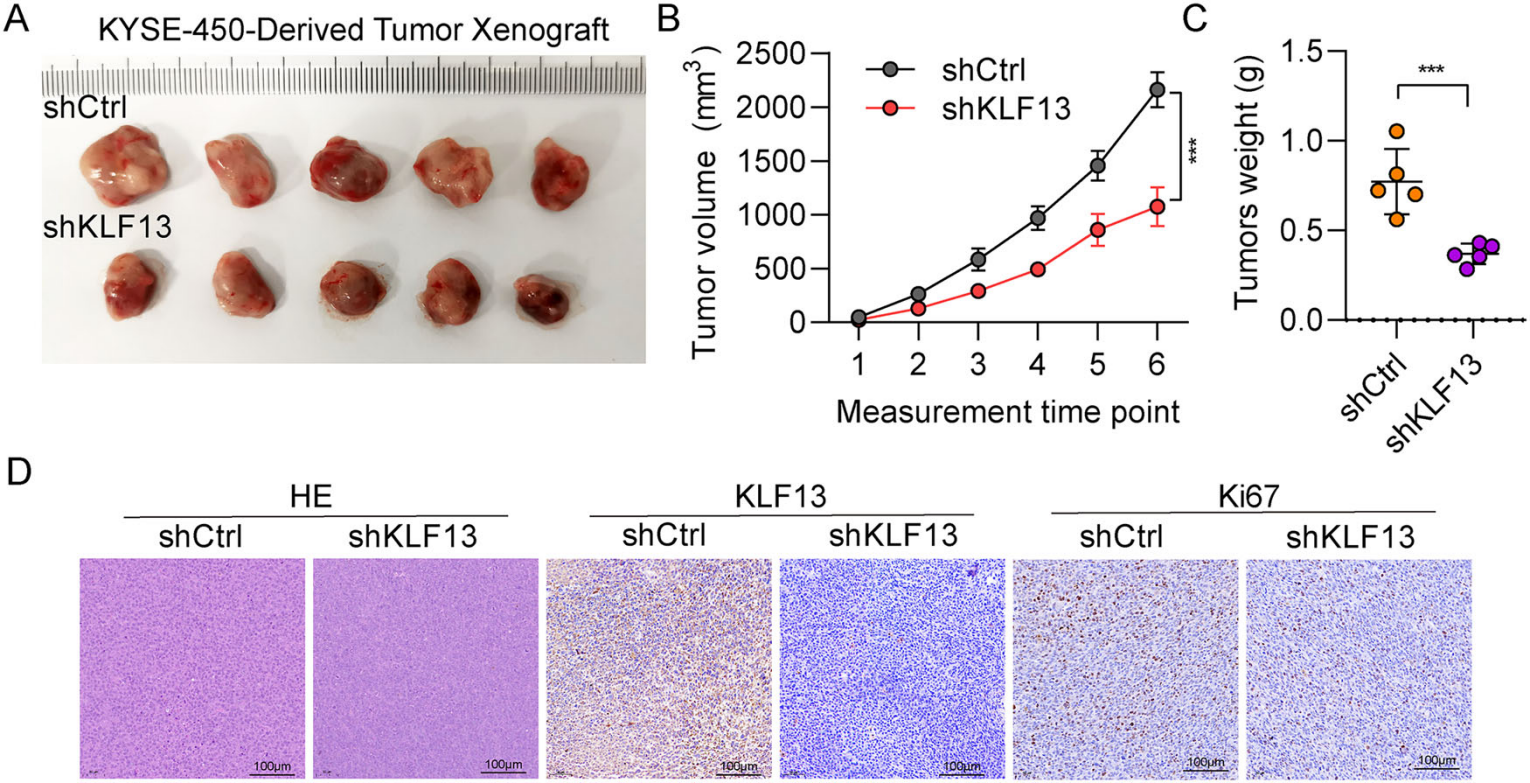
KLF13 silencing suppressed EC tumor growth. **A** Comparison of tumor sizes between the shKLF13 and shCtrl groups. **B**, **C** Comparison of tumor volumes and weights of mice (*n* = 5 per group) from the shKLF13 and shCtrl groups. **D** Ki67 and KLF13 expression in xenograft tumors assessed by IHC analysis. Scale bar = 100 µm.

KYSE-450 cell lines with stable KLF13 knockdown were constructed using KLF13 specific shRNA. KYSE-450 cells were then subcutaneously transplanted into nude mice. KLF13-depleted xenografts generated tumors of smaller volume than the control ones (Fig. 4A). Compare with control group, KLF13-knockdown group had lower tumor volume and weight (Fig. 4B, C). The proliferative marker, Ki67, has weaker IHC staining in tumors treated with KLF13 shRNA (Fig. 4D). These findings further indicate that KLF13 functions as an oncogene in EC.

### KLF13 regulates TG and FFA metabolism

**Fig. 5:**
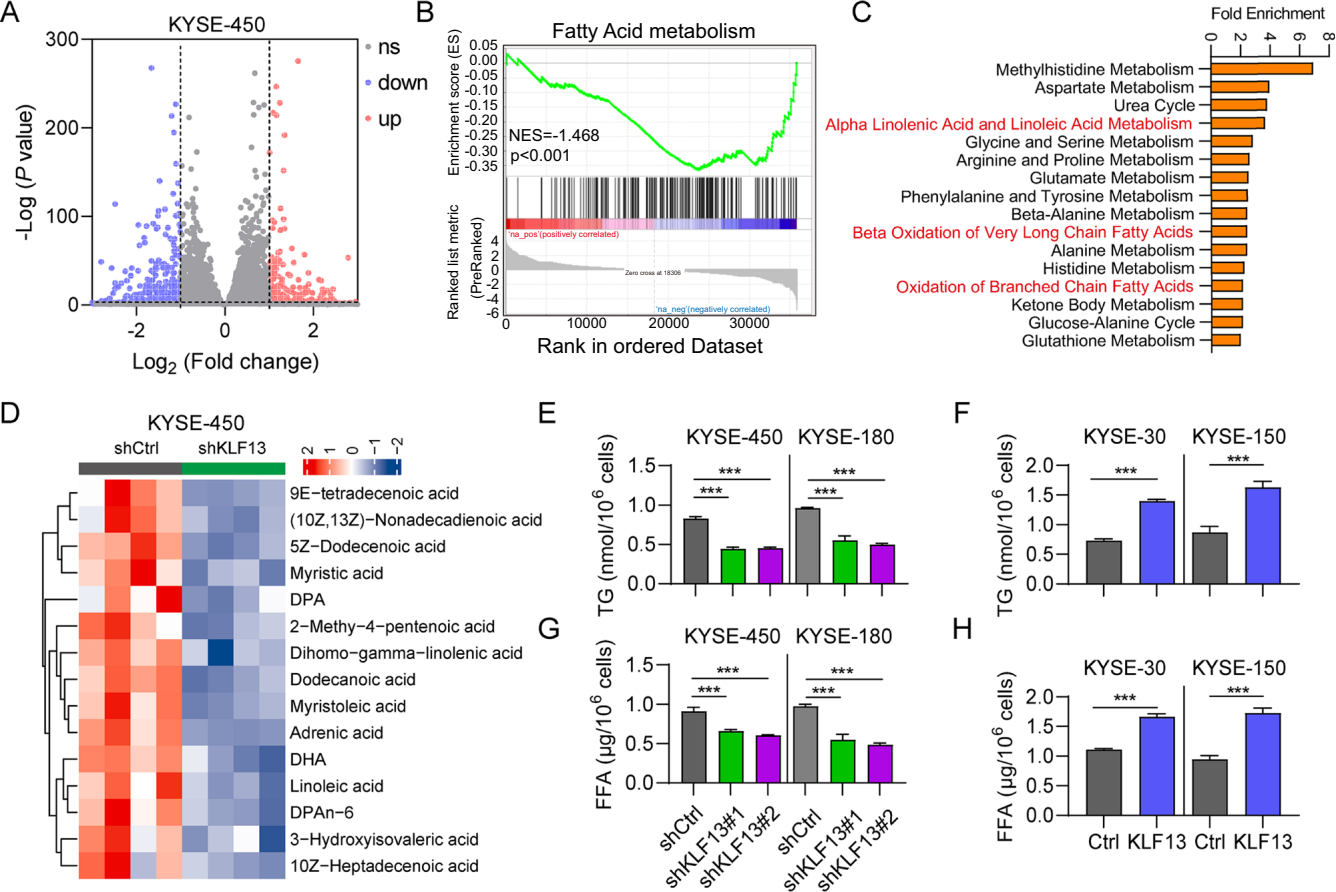
KLF13 regulates lipid metabolism. **A** Volcano plot of differential gene expression in EC from WT and KLF13 knockdown cells using RNA-seq. **B** GSEA plots showing that the fatty-acid metabolism pathway was enriched in the KLF13 knockdown group. **C**, **D** Metabolomics analysis showing that lipid metabolism was significantly altered after KLF13 knockdown. **E**, **F** TG and FFA secretion in the supernatants of KYSE-450 and KYSE-180 cells after KLF13 knockdown. **G**, **H** TG and FFA secretion in the supernatants of KYSE-30 and KYSE-150 cells transfected with Ctrl and KLF13.

RNA-seq analysis was conducted to screen for differentially genes or pathways in KLF13 knockdown cells (Fig. 5A). GSEA results showed that fatty-acid metabolism genes were highly enriched in the KLF13-knockdown EC cells (Fig. 5B). We also conducted metabolomics analysis, which demonstrated that fatty-acid metabolism was mostly decreased after KLF13 knockdown (Fig. 5C, D). Furthermore, we found that TG and FFA contents were markedly reduced in KLF13-knockdown KYSE-450 and KYSE-180 cells (Fig. 5E, F), whereas they were greatly increased following KLF13 overexpression in KYSE-30 and KYSE-150 cells (Fig. 5G, H). These results demonstrate that KLF13 can regulate TG and FFA metabolism in EC cells.

### KLF13 directly bound to *GPIHBP1* and regulated its transcription

**Fig. 6:**
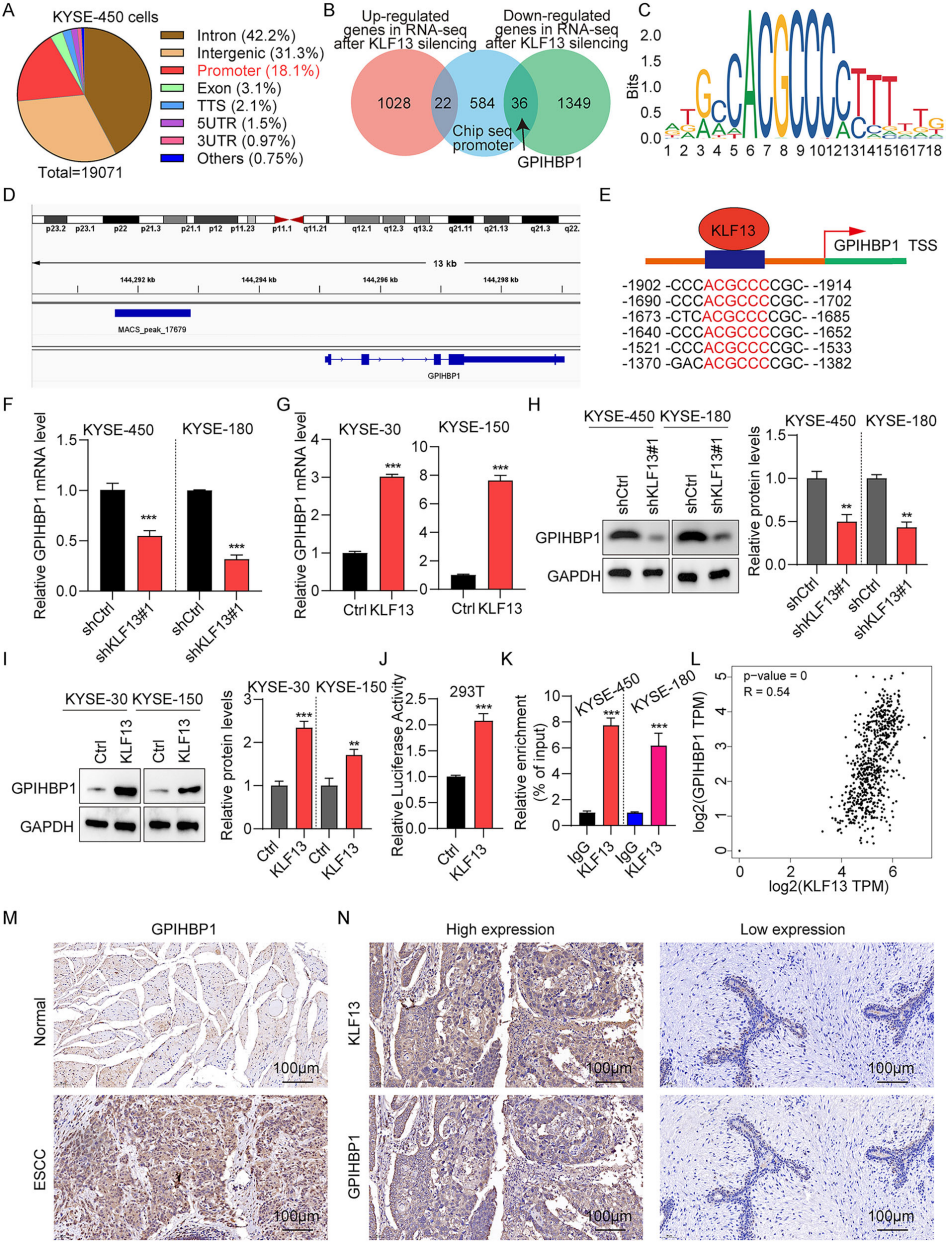
KLF13 directly binds to *GPIHBP1* and regulates its transcription. **A** Pie chart showing Chip-seq promoter results, illustrating the KLF13 binding region. **B** Venn diagram of genes overlapped between the RNA-seq and Chip-seq promoter results. **C** The motif sequence of KLF13. **D** ChIP-seq peak in the *GPIHBP1* promoter region of the human genome. **E** Predicted KLF13 binding site in the *GPIHBP1* promoter region. **F**–**I** GPIHBP1 expression after KLF13 knockdown or KLF13 overexpression in EC cells. **J** KYSE-450 cells were co-transfected with KLF13 overexpressing plasmids, as well as control and luciferase reporter plasmids containing the *GPIHBP1* promoter. **K** KLF13 ChIP experiments (IgG as an internal control). **L** Association between *KLF13* and *GPIHBP1* expression using TCGA samples. **M** GPIHBP1 immunohistochemistry results in EC and normal tissue samples. Bar = 100 μm. **N** Immunohistochemistry results for GPIHBP1 in KLF13 high- or low-EC tissue samples. Bar = 100μm.

To identify downstream target genes of KLF13, we carried out a Chip-seq assay in KLF13 knockdown KYSE-450 cells (Fig. 6A). Genes that overlapped between the results from RNA-seq and Chip-seq assays were identified using a Venn diagram (Fig. 6B). Among them, *GPIHBP1* attracted our attention because it is a GPI-anchored protein necessary for lipoprotein lipase (LPL)-mediated processing of triglyceride-rich lipoproteins. The gene motif of KLF13 was presented in Fig. 6C, and ChIP-seq promoter analysis revealed a clear peak in the promoter of *GPIHBP1* (Fig. 6D). We also scanned the region upstream of *GPIHBP1*, which revealed six potential KLF13 binding sites (Fig. 6E). Furthermore, we found that KLF13 knockdown suppressed GPIHBP1 expression in KYSE-30 and KYSE-150 cells, whereas KLF13 overexpression increased GPIHBP1 expression (Fig. 6F–I). Moreover, KLF13 overexpression increased the luciferase activity in KYSE-450 cells, indicating an interaction between KLF13 and *GPIHBP1* (Fig. 6J). Furthermore, ChIP-PCR results demonstrated binding of KLF13 to the *GPIHBP1* promoter (Fig. 6K). A positive association (*R* = 0.54) between the expression of KLF13 and GPIHBP1 was also discovered by analysis of TCGA data (Fig. 6L). Similarly, GPIHBP1 protein levels were higher in EC tissues than normal specimens (Fig. 6M, Table S2). IHC results showed a positive relation between the expression of GPIHBP1 and KLF13 in tumor samples (Fig. 6N, Table S3). The effect of GPIHBP1 on EC cell proliferation and migration were also discussed, with GPIHBP1 knockdown decreased cell proliferation and migration (Fig. S2A–D), while GPIHBP1 overexpression increased EC cell proliferation and migration (Fig. S2E–H). These results suggested that KLF13 interacted with *GPIHBP1* and regulates its levels of transcription levels, and GPIHBP1 promoted EC cell proliferation and migration.

### KLF13 regulated EC cell proliferation, tumor growth, and TG and FFA metabolism through GPIHBP1

**Fig. 7:**
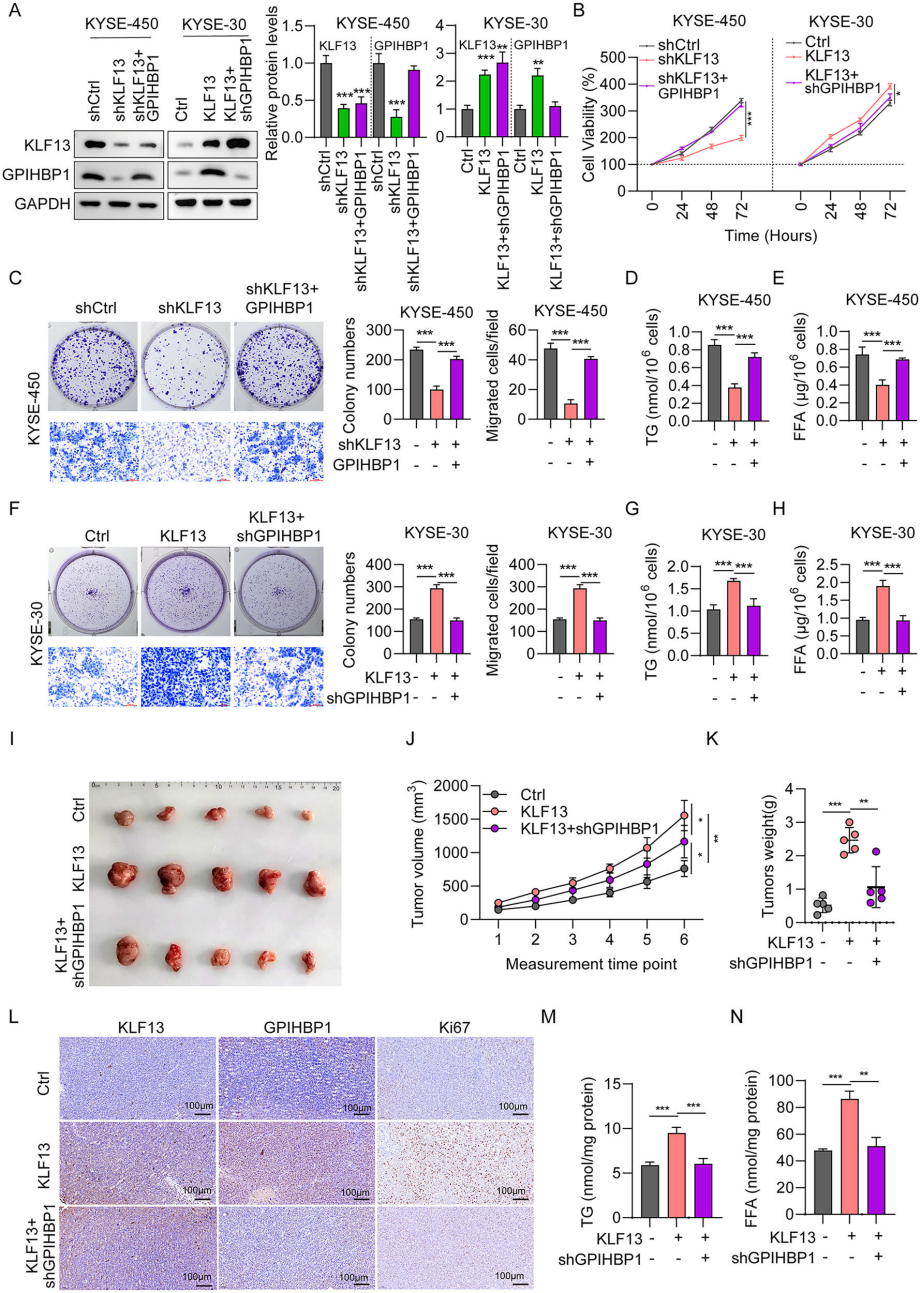
KLF13 regulates EC cell proliferation, tumor growth, and lipid metabolism through GPIHBP1. **A** KLF13 and GPIHBP1 protein levels in KYSE450 cells transfected with shCtrl, shKLF13, and shKLF13 +GPIHBP, and KYSE30 cells transfected with Ctrl, KLF13, and KLF13+ shGPIHBP. **B** Cell viability of KYSE450 and KYSE30 cells. **C**, **D** Cell colony numbers of KYSE450 and KYSE30 cells. **E**–**H** TG and FFA concentrations in KYSE450 and KYSE30 cells. **I** Comparison of tumor sizes between the Ctrl, KLF13, and KLF13+shGPIHBP groups. **J**, **K** Comparison of tumor volumes and weights in the Ctrl, KLF13 and KLF13 + shGPIHBP groups (*n* = 5 mice per group). **L** KLF13, GPIHBP, and Ki67 expression in xenograft tumors evaluated by H&E staining. Scale bar = 100 µm. **M**, **N** TG and FFA concentrations in tumors from Ctrl, KLF13, and KLF13+shGPIHBP group mice.

Next, we conducted recovery assays to confirm the roles of KLF13 and GPIHBP1 in EC progression. GPIHBP1 overexpression plasmids were transfected into KYSE450 cells to rescue GPIHBP1 expression after KLF13 knockdown, or shGPIHBP1 was introduced into KLF13-overexpressing KYSE-30 cells. GPIHBP1 protein expression was restored in KLF13 knockdown cells (Fig. 7A), while cell viability was also restored in shKLF13 + GPIHBP1 EC Cells compare to shKLF13 cells (Fig. 7B). Further, shGPIHBP1 knockdown reduced the increased GPIHBP1 protein levels in response to KLF13 overexpression (Fig. 7A), and restored cell viability to normal levels in KLF13 + shGPIHBP1 KYSE-30 cells (Fig. 7B). Furthermore, GPIHBP1 overexpression also restored cell colony formation ability, as well as TAG and FFA content, which were suppressed by shKLF13, in KYSE-450 cells (Fig. 7C–E). Conversely, shGPIHBP1 reduced cell colony numbers, as well as TAG and FFA content, in KYSE-30 cells, which were induced following KLF13 overexpression (Fig. 7F–H).

Experiments using an EC xenograft model demonstrated that the oncogenic role of KLF13 was mediated through regulation of GPIHBP1, as tumor size (Fig. 7I), tumor volume (Fig. 7J) and tumor weight (Fig. 7K) were all greatly reduced in KLF13 + shGPIHBP1 group relative to those in KLF13 group. IHC staining of tumor tissues showed that KLF13 and GPIHBP1 expression were increased in KLF13 overexpressing mice, while they were decreased in mice with GPIHBP1 knocked down (Fig. 7L). TAG and FFA concentrations in tumor tissues from the KLF13 + shGPIHBP1 group were markedly lower compare to KLF13 group (Fig. 7M, N). These data revealed that KLF13 regulates EC cell proliferation, tumor growth, and lipid metabolism through regulating GPIHBP1 expression.

## Discussion

Human *KLF13* cDNA was reported by Song et al. in 1999 [28], and its modular structure was demonstrated in 2002 [29]. KLF13 was first reported as a tumor promoting in 2010 in oral squamous cell carcinoma [30]. KLF13 may be a tumor suppressor or promoter is depending on the tissue. For example, KLF13 acts as a tumor suppressor in colorectal cancer [17], gastric cancer [31], glioma [32], pancreatic cancer [18], and prostate cancer [33]. KLF13 function even varies among different cell types in the same cancer. For example, KLF13 knockdown led to decreased apoptosis and increased proliferation of hepatocellular carcinoma (HCC) cells in vitro [34]; however, in another study, KLF13 was found to be over-expressed in HCC tissue, while KLF13 knockdown suppressed HCC cell proliferation, migration, and invasion, and drove HCC cell apoptosis in vitro [35]. KLF13 was reported to be upregulated in cervical cancer [36], but downregulated in colorectal cancer [17, 37], while reduced KLF13 expression in non-small cell lung cancer tumours indicated poor overall survival [19].

We also found high KLF13 expression in EC tissues and cells in this study, and that high KLF13 expression correlated with poor prognosis of patient. Our functional studies showed that KLF13 knockdown decreased cell viability, promoted apoptosis and induced cell cycle arrest of EC cells, thereby suppressing tumor growth, whereas KLF13 overexpression drove EC cell proliferation and migration, indicating a tumor promoter role for KLF13 in EC.

Aberrant lipid metabolism and metabolic reprogramming constitute significant biological hallmarks of malignant tumors and exhibit a strong association with the onset and advancement of esophageal cancer by promoting cancer cells uncontrolled proliferation, survival, invasion, and resistance to antineoplastic therapy. Metabolomics has become a new platform for biomarker discovery over recent years [38]. Dysregulation of lipid metabolism is common in cancer [39]. Lipids form a diverse group of water-insoluble molecules, such as TAG, which exert significant effects at the cell and organism levels. Fatty acids are the main building blocks for TAG synthesis, which is primarily used for energy storage [40]. In EC, enzymes involved in lipid metabolism promote cell metastasis [41]. Parthasarathi et al. identified 487 lipid metabolism-related genes in patients with EC [42]. Further, Shen et al. constructed a prognostic model based on a 4-gene signature associated with lipid metabolism, which was effective for predicting prognosis among patients with EC [43]. KLF13 is known to intersect with adipogenesis function [44], and promotes adipocyte differentiation [45]. Our RNA-seq results show that fatty-acid metabolism is an enriched process in EC cells, and we confirmed that some lipids were significantly downregulated after KLF13 knockdown. We also detected TAG and FFA levels in EC cells and found that they were significantly decreased after KLF13 knockdown and greatly increased following KLF13 overexpression.

Furthermore, Chip-seq promoter assay was used to explore the downstream genes of KLF13 in EC, as it was a transcription factor. Combined with the results of Chip-seq and RNA-seq, a lipid-related gene, glycosylphosphatidylinositol-anchored high-density lipoprotein-binding protein 1 (GPIHBP1), was identified. GPIHBP1 removes LPL from interstitial spaces to the capillary lumen [46], where LPL has key functions in normal lipid metabolism [47]. GPIHBP1 has an N-terminal acidic domain and a cysteine-rich Ly6 domain [48, 49]. The acidic domain is highly enriched in aspartic and glutamic acid residues and mediates the binding of LPL and Apo-AV [50]. The Ly-6 protein domain contains 10 conserved cysteine residues, is approximately 80 amino acids long, and has a defined disulfide-bonding pattern [51]. In this study, we found several KLF13 binding sites in the promoter region of the *GPIHBP1* gene, while RT-PCR and western blot analyses confirmed that GPIHBP1 levels altered in response to KLF13. Chip-PCR and luciferase assay also demonstrated binding between KLF13 and *GPIHBP1*. The expression of KLF13 was positively correlated with GPIHBP1 expression by analysis of data from TCGA database. IHC showed that high GPIHBP1 expression in EC tissues was consistent with KLF13 expression.

GPIHBP1 is reported to be downregulated in breast cancer relative to precancerous tissues [52], and to facilitate TRL processing in glioma cells [53]. Our functional research demonstrated that GPIHBP1 knockdown suppressed EC cell proliferation and migration, whereas GPIHBP1 overexpression promoted EC progression. Finally, we conducted recovery assays to assess whether KLF13 exerted its oncogenic role through activating GPIHBP1. Two set cell experiments, with “KLF13 knockdown + GPIHBP1 overexpression” and “KLF13 overexpression + GPIHBP1 knockdown”, demonstrated the role of KLF13 in promoting EC cell proliferation, migration, and lipid regulation recovery to normal levels through suppression of GPIHBP1, and increased tumour size in vivo. Conversely, the suppressive effect on EC cell progression induced by KLF13 knockdown could be ameliorated by GPIHBP1 overexpression.

In summary, KLF13 is upregulated in EC tissue and cells, and that high KLF13 expression indicated poor prognosis. Further, we reveal that KLF13 functions as an oncogene that promotes EC progression and regulates lipid metabolism. Mechanistically, KLF13 exerts its oncogenic role through transcriptional activation of its downstream target, GPIHBP1.

## Supplementary information

WB org

Supplementary figure and table

## Data Availability

The datasets used and/or analyzed during the current study are available from the corresponding author on reasonable request.
