# Supplementary figures and images for: KLF13 promotes esophageal cancer progression and regulates triacylglyceride and free fatty acid metabolism through GPIHBP1
Source: Cell Death Dis. 2025 May 31;16(1):425. doi: 10.1038/s41419-025-07709-7 (PMC12126484; doi:10.1038/s41419-025-07709-7)

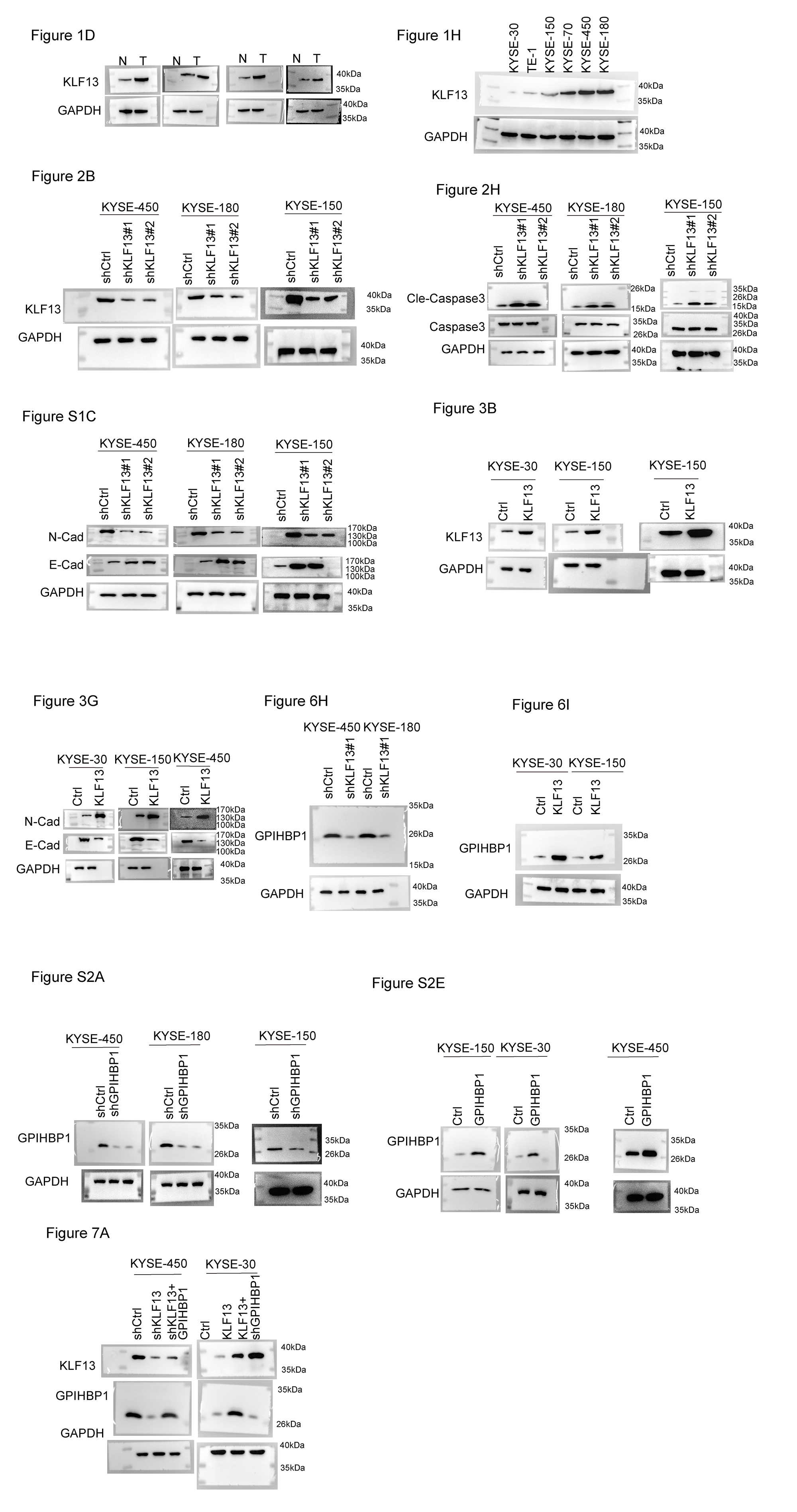

Supplement: Supplementary file 1 — WB org [file 41419_2025_7709_MOESM1_ESM.tif]
