# Supplementary material for: KLF13 promotes esophageal cancer progression and regulates triacylglyceride and free fatty acid metabolism through GPIHBP1
Source: Cell Death Dis. 2025 May 31;16(1):425. doi: 10.1038/s41419-025-07709-7 (PMC12126484; doi:10.1038/s41419-025-07709-7)
Supplement: Supplementary file 2 — Supplementary figure and table [file 41419_2025_7709_MOESM2_ESM.docx]

**Supplementary files**

**
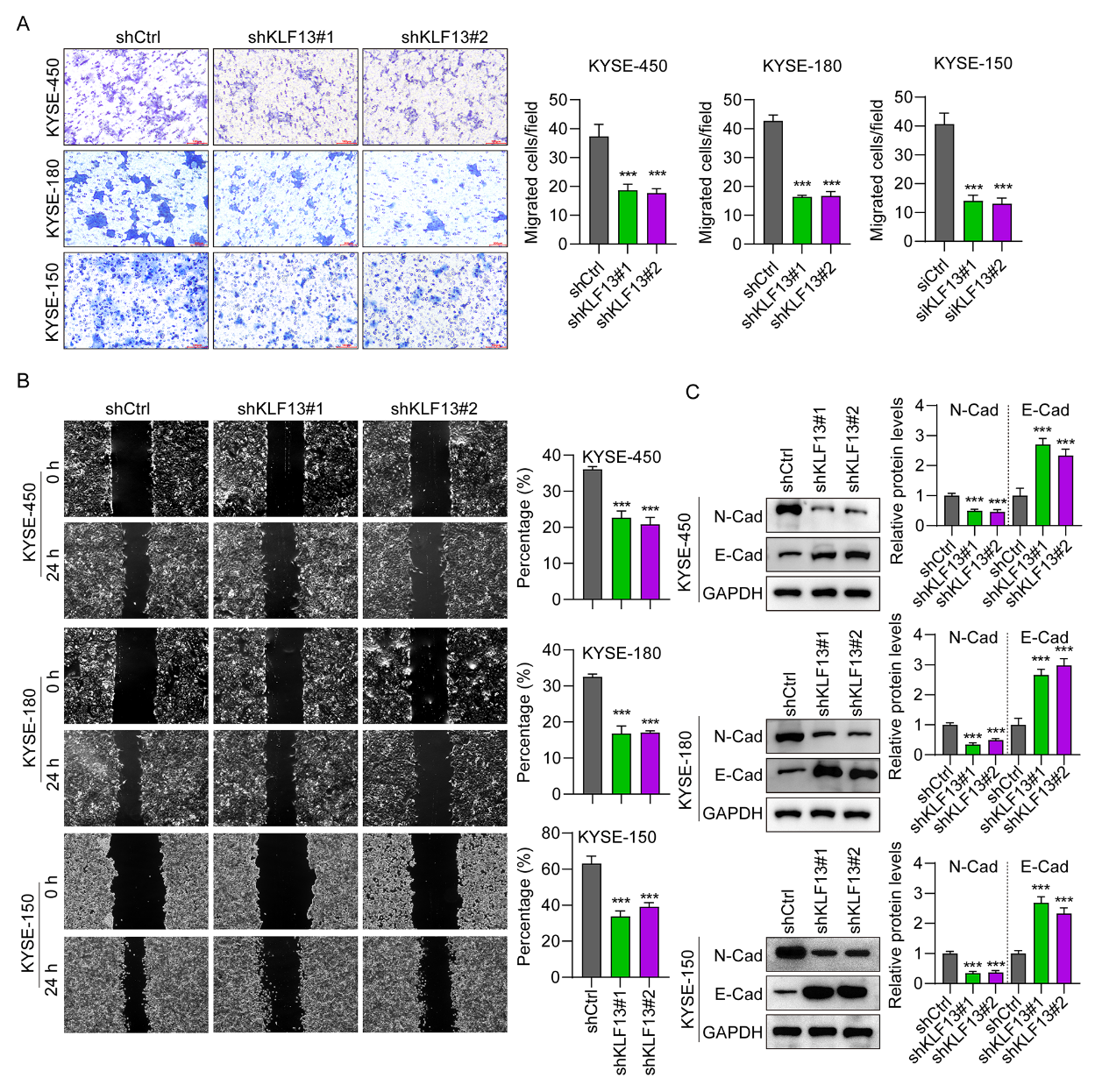
**

**Fig. S1** KLF13 silencing suppressed EC cell migration. **A** Transwell assays to investigate cell migration of three EC cell lines transfected with shKLF13#1, shKLF13#2, and shCtrl. **B** Wound healing assays to investigate migration of three EC cell lines transfected with shKLF13#1, shKLF13#2, and shCtrl. **C** Western blot analysis of E-cadherin and N-cadherin levels in EC cells transfected with shKLF13#1, shKLF13#2, and shCtrl.


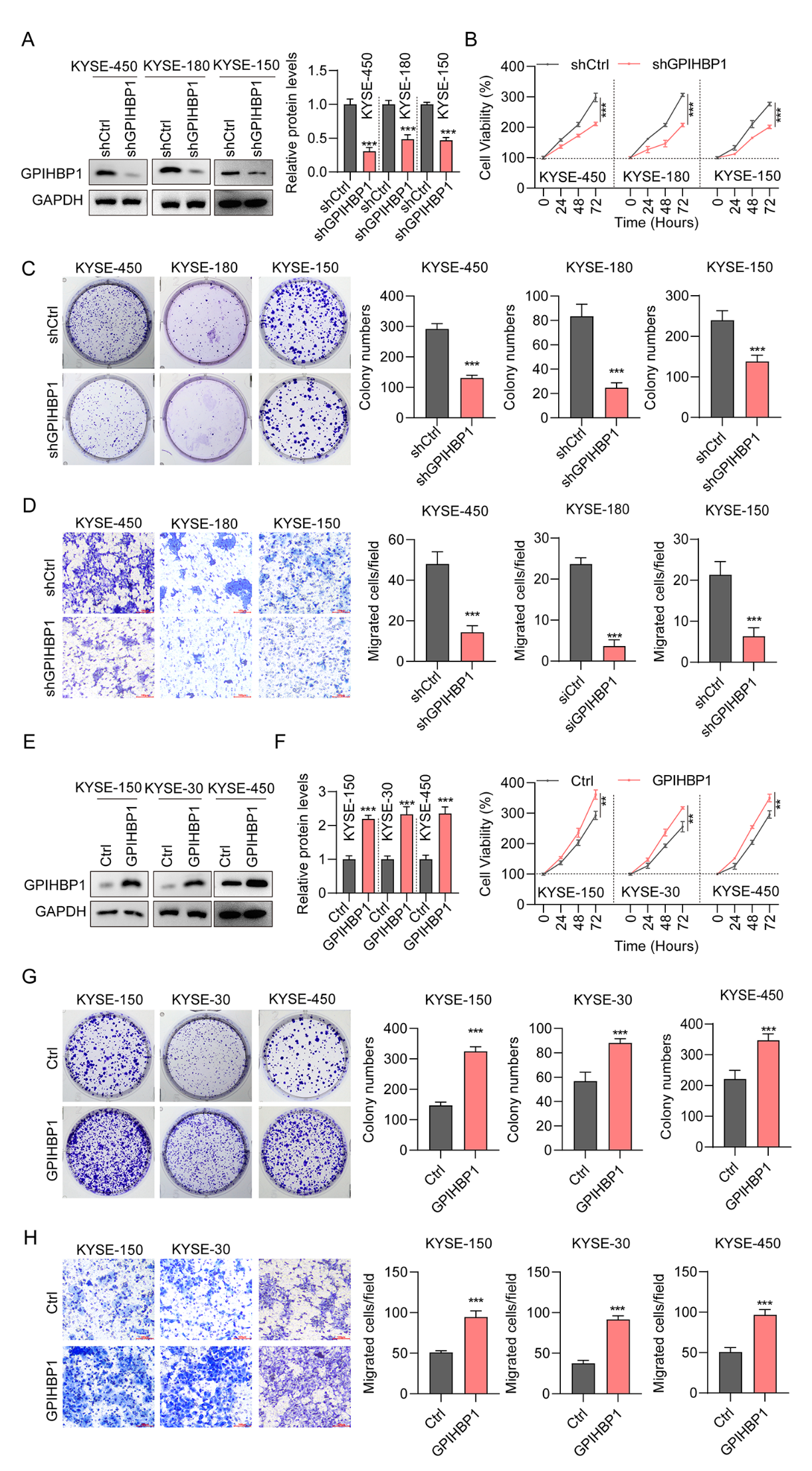


**Fig. S2** GPIHBP1 drives esophageal cancer proliferation and metastasis. **A** GPIHBP1 expression in EC cells (KYSE450, KYSE180 and KYSE150) infected with shCtrl and shGPIHBP. **B** Cell viability of EC cells infected with shCtrl and shGPIHBP1. **C** Colony formation assays to assess KYSE450, KYSE180 and KYSE150 cell colony formation ability after transfection with shCtrl and shGPIHBP1. **D** Transwell assays of KYSE450, KYSE180 and KYSE150 cells transfected with shCtrl and shGPIHBP1. **E** GPIHBP1 protein levels in EC cells (KYSE450, KYSE180 and KYSE150) infected with Ctrl and GPIHBP, detected by western blot. **F** CCK-8 assays to assess the relative viability of EC cells transfected with Ctrl and GPIHBP1. **G** Cell colony numbers in EC cells infected with Ctrl and GPIHBP1. **H** Cell migration ability of EC cells infected with Ctrl and GPIHBP1.

**Table S1** Correlation of KLF13 expression with clinicopathological characteristics in 100 patients of ESCC.

|  | Characteristic | KLF13 expression | | p Value |
| --- | --- | --- | --- | --- |
|  |  | High | Low |  |
| Age | <60 | 17 | 12 | 0.474 |
|  | ≥60 | 47 | 24 |  |
| Gender | M | 42 | 24 | 0.24 |
|  | F | 12 | 12 |  |
| Tumor size (cm) | <5 | 20 | 20 | 0.017 |
|  | ≥5 | 44 | 16 |  |
| Pathology grade | I-II | 54 | 32 | 0.532 |
|  | III-IV | 10 | 4 |  |
| Number of lymph nodes | <7 | 32 | 15 | 0.579 |
|  | ≥7 | 32 | 19 |  |

**Table S2** The protein abundance of GPIHBP1 in ESCC by IHC.

|  | Normal | Cancer | χ^2^ | p Value |
| --- | --- | --- | --- | --- |
| High expression | 22 | 66 | 26.36 | <0.001 |
| Low expression | 58 | 34 |  |  |
| Total | 80 | 100 |  |  |

**Table S3** Spearman correlation analysis of expression between KLF13 and GPIHBP1 in 100 ESCC tissues by IHC.

|  | KLF13 | |  |
| --- | --- | --- | --- |
|  | *r_s_* | *P* value |  |
| GPIHBP1 | 0.306 | 0.002 |  |

r, Spearman correlation
